# Comparative interactomics analysis reveals potential regulators of α6β4 distribution in keratinocytes

**DOI:** 10.1242/bio.054155

**Published:** 2020-08-13

**Authors:** Lisa te Molder, Liesbeth Hoekman, Maaike Kreft, Onno Bleijerveld, Arnoud Sonnenberg

**Affiliations:** 1 Division of Cell Biology I, The Netherlands Cancer Institute, Plesmanlaan 121, Amsterdam 1066 CX, The Netherlands; 2 Mass Spectrometry/Proteomics Facility, The Netherlands Cancer Institute, Plesmanlaan 121, Amsterdam 1066 CX, The Netherlands

**Keywords:** Integrin, Interactome, BioID, Hemidesmosome, Cortical microtubule stabilizing complex, Tetraspanin

## Abstract

The integrin α6β4 and cytoskeletal adaptor plectin are essential components of type I and type II hemidesmosomes (HDs). We recently identified an alternative type II HD adhesion complex that also contains CD151 and the integrin α3β1. Here, we have taken a BioID proximity labeling approach to define the proximity protein environment for α6β4 in keratinocytes. We identified 37 proteins that interacted with both α6 and β4, while 20 and 78 proteins specifically interacted with the α6 and β4 subunits, respectively. Many of the proximity interactors of α6β4 are components of focal adhesions (FAs) and the cortical microtubule stabilizing complex (CMSC). Though the close association of CMSCs with α6β4 in HDs was confirmed by immunofluorescence analysis, CMSCs have no role in the assembly of HDs. Analysis of the β4 interactome in the presence or absence of CD151 revealed that they are strikingly similar; only 11 different interactors were identified. One of these was the integrin α3β1, which interacted with α6β4 more strongly in the presence of CD151 than in its absence. These findings indicate that CD151 does not significantly contribute to the interactome of α6β4, but suggest a role of CD151 in linking α3β1 and α6β4 together in tetraspanin adhesion structures.

## Abstract (precis)

**Summary:** Comparative interactomics analysis reveals close proximity of HDs, FAs and CMSCs, and a role of CD151 in linking α3β1 and α6β4 together in an alternative type II HD-like adhesion complex.

## INTRODUCTION

The cytoskeleton of epithelial cells is an integrated network of actin microfilaments, keratin intermediate filaments and microtubules, which helps the cell to maintain its shape and internal organization. The actin and keratin networks are linked to the cytoskeletons of adjacent cells and the extracellular matrix by specialized junctional complexes and, thereby, contribute to tissue integrity and intracellular communication (Kirfel et al., 2003; Blanchoin et al., 2014). While the microtubule cytoskeleton is responsible for the intracellular transport of membrane-bound vesicles and organelles.

Hemidesmosomes (HDs) are integrin-based adhesion complexes that mediate stable anchorage of epithelial cells to the underlying basement membrane and serve as anchoring sites for the keratin cytoskeleton to the plasma membrane. Two types of HDs can be distinguished according to their protein composition. The integrin α6β4, a heterodimeric transmembrane protein, is the main component of both type I and type II HDs (Uematsu et al., 1994; Fontao et al., 1997; Sterk et al., 2000; Litjens et al., 2006; Walko et al., 2015). In addition to α6β4, these two structures share the tetraspanin CD151 and the cytoskeletal linker protein plectin, which through binding to β4 and keratin establishes a linkage between the extracellular matrix (ECM) and the keratin intermediate filament (IF) cytoskeleton. In type I HDs an additional linkage between the plasma membrane and keratin filaments through BP180 and BP230 enhances the stability of these adhesion structures. A third CD151-containing adhesion structure formed in the central region of cultured keratinocytes contains the integrin α3β1 in addition to the type II HD components α6β4 and plectin (te Molder et al., 2019). Absence or defects of HD proteins compromises epithelial integrity, which results in a blistering disorder called Epidermolysis Bullosa (Has and Bruckner-Tuderman, 2014). Besides its role in mediating stable cell-matrix adhesion, the HD integrin α6β4 cooperates with growth factor receptors to promote pro-tumorigenic signaling (Ramovs et al., 2017).

Linkage of the actin cytoskeleton to the extracellular matrix occurs (amongst others) at integrin-based multiprotein complexes termed focal adhesions (FAs). The molecular composition of these adhesions may vary depending on external cues and cellular responses (Horton et al., 2015). However, their core always consists of integrins and adaptor proteins such as talin and vinculin. In addition to providing adhesion of the cells, FAs are essential for cell migration.

In close proximity of FAs, cytoplasmic linker associated proteins (CLASPs) anchor the plus ends of microtubules to the plasma membrane through a complex of LL5α/β (also known as PHLDB1/2), liprin α1/β1 and ELKs (also known as ERC1) (Mimori-Kiyosue et al., 2005; Lansbergen et al., 2006; Akhmanova and Steinmetz, 2008). KANK proteins have been identified as the adaptor proteins that can link this complex of proteins, known as the cortical microtubule stabilizing complexes (CMSCs), to FAs. They bind on the one hand to talin in FAs and on the other hand to liprin β1 in the CMSCs. By serving as sites for the targeted delivery of microtubule bound vesicles containing MMPs at the cell membrane, the CMSCs are thought to play an important role in the turnover of FAs and actin reorganization (Rodriguez et al., 2003; Akhmanova et al., 2009; Stehbens et al., 2014; Dogterom and Koenderink, 2019).

Recently, we showed that FAs and HDs are mechanically coupled, and that plectin, which binds integrin β4 and F-actin in a mutually exclusive manner, plays a central role in this coupling (Wang et al., 2020). In this study, we used proximity dependent biotinylation (BioID) (Roux et al., 2012) to map the interactomes of the integrin α6 and β4 subunits in keratinocytes and assessed the contribution of CD151 to the β4 interactome. Furthermore, we investigated the role of CMSC components in HD dynamics.

## RESULTS

### Identification of the β4 associated proteins by proximity-dependent biotinylation

**Fig. 1.**
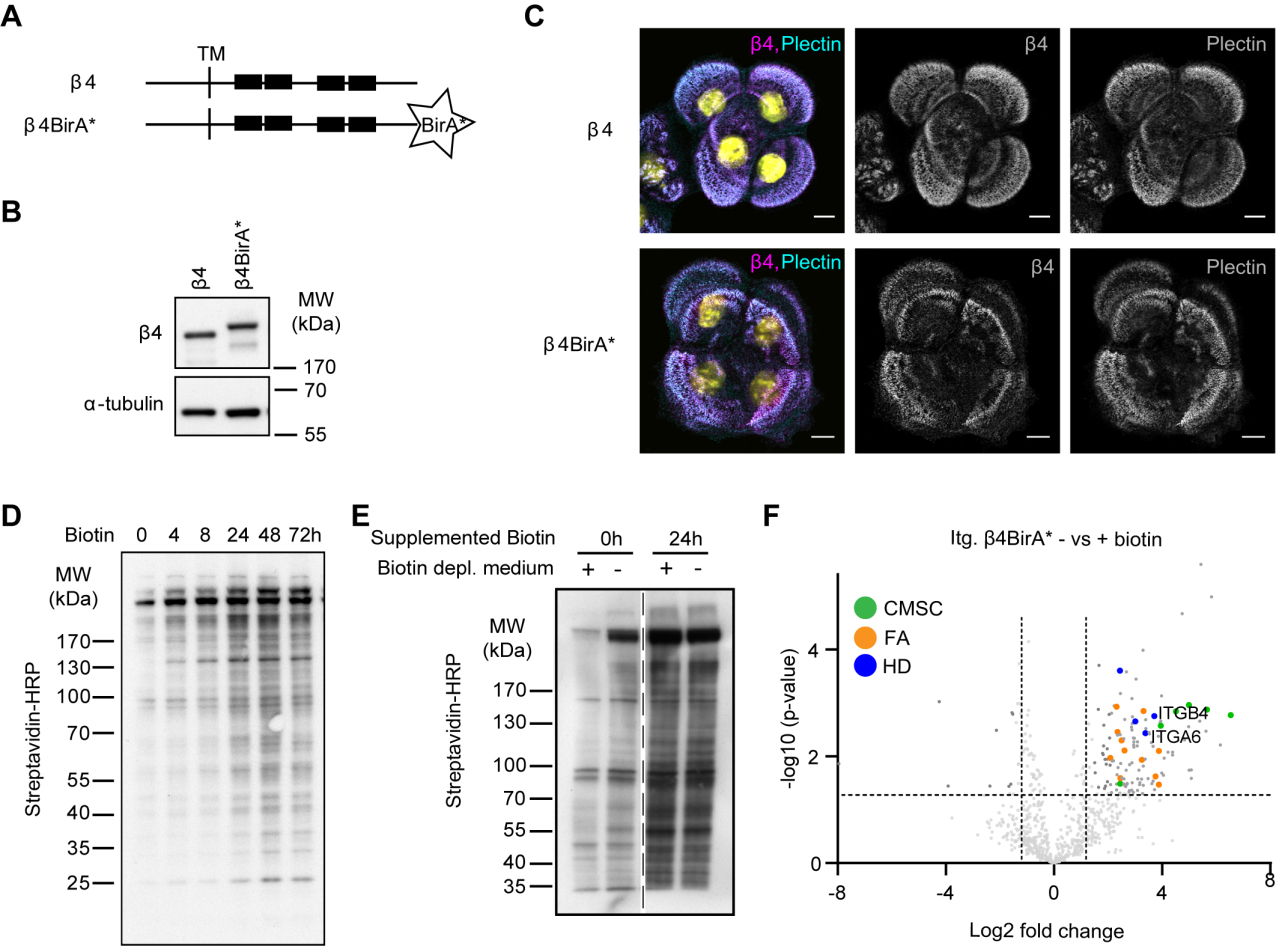
**BioID method to identify β4 proximity interactors.** (A) Schematic representation of wild-type β4 and the β4-BirA* fusion proteins. Black boxes indicate the FnIII domains. TM is transmembrane domain. (B) Western blot analysis of whole cell lysates from PA-JEB/β4 and PA-JEB/β4-BirA* keratinocytes probed with anti-β4 and anti-α-tubulin (loading control) antibodies. (C) Representative confocal microscopy images of PA-JEB keratinocytes expressing wild-type β4 and β4-BirA* stained for β4 and plectin. Scale bars: 10 μm. (D) Whole cell lysates from PA-JEB/β4-BirA* keratinocytes treated for the indicated time points with 50 μM biotin and analyzed by western blot with streptavidin-HRP. (E) Western blot analysis of biotinylated proteins from PA-JEB/β4-BirA* cells, cultured in regular medium or biotin-depleted medium for 20 h and subsequently treated with or without biotin for 24 h. Biotinylated proteins were detected by probing the membrane with Streptavidin-HRP. (F) Volcano plot showing enrichment (log_2_) and corresponding significance (*P*-value, log_10_) of biotinylated proteins in biotin-treated and -untreated PA-JEB/β4-BirA* keratinocytes (*n*=3).

To identify proteins that interact with integrin α6β4 in keratinocytes, we applied the BioID method (Roux et al., 2012), which has been successfully used to identify proteins that reside in close proximity of a specific protein of interest, including proteins that are only transiently associated (van Itallie et al., 2013, 2014; Fredriksson et al., 2015; Dong et al., 2016). BioID employs a modified biotin ligase BirA* fused to a protein of interest that biotinylates proteins in close proximity (20 nm) upon addition of biotin. We fused BirA* to the C-terminal tail of full-length integrin β4 (Fig. 1A) and stably expressed the fusion protein in β4-deficient PA-JEB keratinocytes by retroviral transduction. Expression of the β4-BirA* fusion protein (225 kDa) was confirmed by western blot (Fig. 1B). Like wild-type β4, the fusion protein localized in HDs as visualized by immunofluorescence (Fig. 1C).

Treatment of the β4-BirA* expressing cells with biotin resulted in a time-dependent increase in biotinylated proteins (Fig. 1D). Notably, a number of proteins were biotinylated in the absence of exogenously added biotin. In an effort to reduce the level of biotinylation of these background proteins, we depleted the culture medium of biotin by treatment with Streptavidin agarose beads, which reduced the amount of background proteins (Fig. 1E).

Biotinylated proteins were collected using streptavidin-Sepharose beads and analyzed by mass spectrometry. As expected, the integrin β4 subunit and its partner subunit α6, were identified among 130 other hits. Other interactors were components of HDs and multiple proteins of two other membrane complexes, CMSCs and FAs (Fig. 1F; Table S1).

### Interactomics analysis of α6β4-associated proteins

**Fig. 2.**
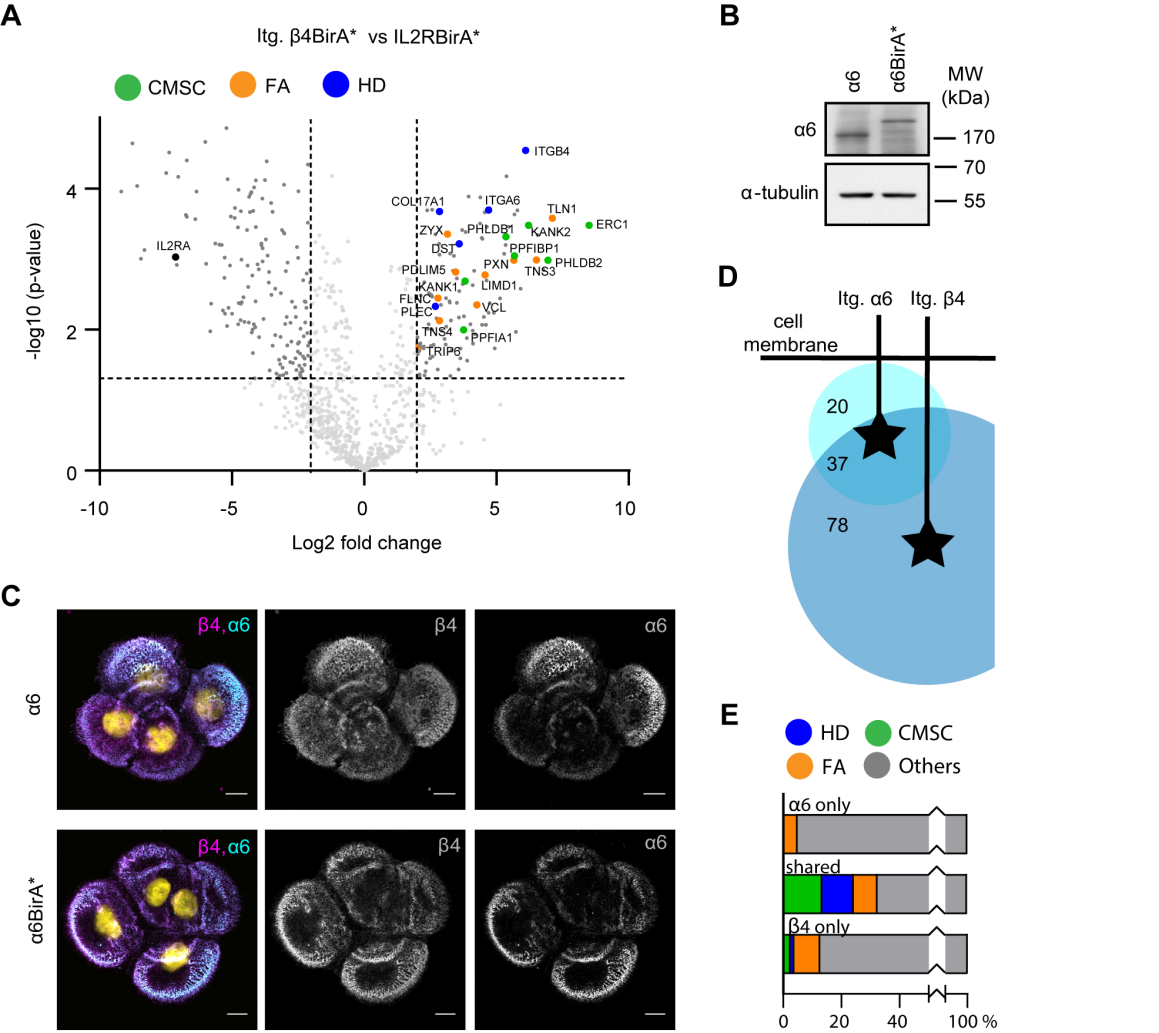
**Interactomics analysis of α6β4 associated proteins.** (A) Volcano plot showing enrichment (log_2_) versus significance (*P*-value, log_10_) of proteins identified by β4-BirA* relative to control IL2R-BirA* (*n*=3). Components of the CSMCs, FAs and HDs are highlighted in green, orange and blue, respectively. (B) Western blot analysis of whole cell lysates from PA-JEB/β4 and PA-JEB/β4 α6-BirA* keratinocytes probed with anti-α6 and anti-α-tubulin (loading control) antibodies. (C) Representative confocal microscopy images of PA-JEB/β4 keratinocytes expressing endogenous α6 or α6-BirA* stained for α6 and β4. Scale bars: 10 μm. (D) Venn diagram depicting the number of overlapping hits between interacting proteins of the Itg. α6 and Itg. β4 subunit. Numbers of overlapping and specific proteins are presented. (E) Bar graphs showing the percentage of HD, CMSC and FA proteins in the α6 and β4 interactomes as well as their percentage among the interactors shared by the two interactomes.

To demonstrate the specificity of the β4 interactions with components of FAs and CMSCs, we performed an additional BioID experiment with an irrelevant transmembrane protein, the interleukin2 receptor (IL2R). Stable cell lines expressing the IL2R fused to BirA* were generated by retroviral infection. The IL2R-BirA* fusion protein was expressed in PA-JEB/β4 keratinocytes. Pairwise comparison of the β4-BirA* and IL2R-BirA* datasets (obtained after treatment of the cells with 50 μM biotin) revealed that 115 proteins interacted specifically with integrin β4. Consistent with the results of the comparison of the β4 samples treated with or without biotin, we identified multiple HD proteins (5/6) and FA proteins (10/60), and all components of the CMSC (7/7) as proximity interactors of β4 (Fig. 2A; Table S2).

Next, we determined the proximity interactors of the integrin α6 subunit. To this end, the α6 subunit fused to BirA* was expressed in PA-JEB/β4 keratinocytes, in which the α6 gene (*ITGA6*) had been disrupted by CRISPR/Cas9 technology. Expression and localization of the α6-BirA* fusion protein in HDs was confirmed by western blot and immunofluorescence analyses (Fig. 2B,C). A total of 57 proximity interactors of α6 were identified that were absent from the negative IL2R-BirA* sample. 37 of these proteins were also identified in the β4 interactome. Again, many of the shared proteins were components of HDs, CMSCs and FAs (Fig. 2E). The analysis also revealed a significant number of subunit-specific interactors, 20 for α6 and 78 for β4 (Fig. 2D). The fact that more proteins were identified with β4-BirA* than with α6-BirA* could be due to flexibility of the unusually long β4 cytoplasmic domain (1019 amino acids compared to 54 amino acids in α6), which allows the fused biotin ligase not only to biotinylate proteins that are spatially located near to but also distant from the α6 subunit. It is important to note that keratinocytes do not express α6β1 and that, therefore, the interactors of α6 subunit can be considered to be specific for α6β4 (Table S3) (Sonnenberg et al., 1991; te Molder et al., 2019).

In conclusion, the interactome of integrin α6β4 in keratinocytes contains 135 proteins, and many of the previously mentioned HD, CMSC and FA proteins were among the significant interactors of both α6 and β4.

### β4 proximity interactors in the presence and absence of CD151

**Fig. 3.**
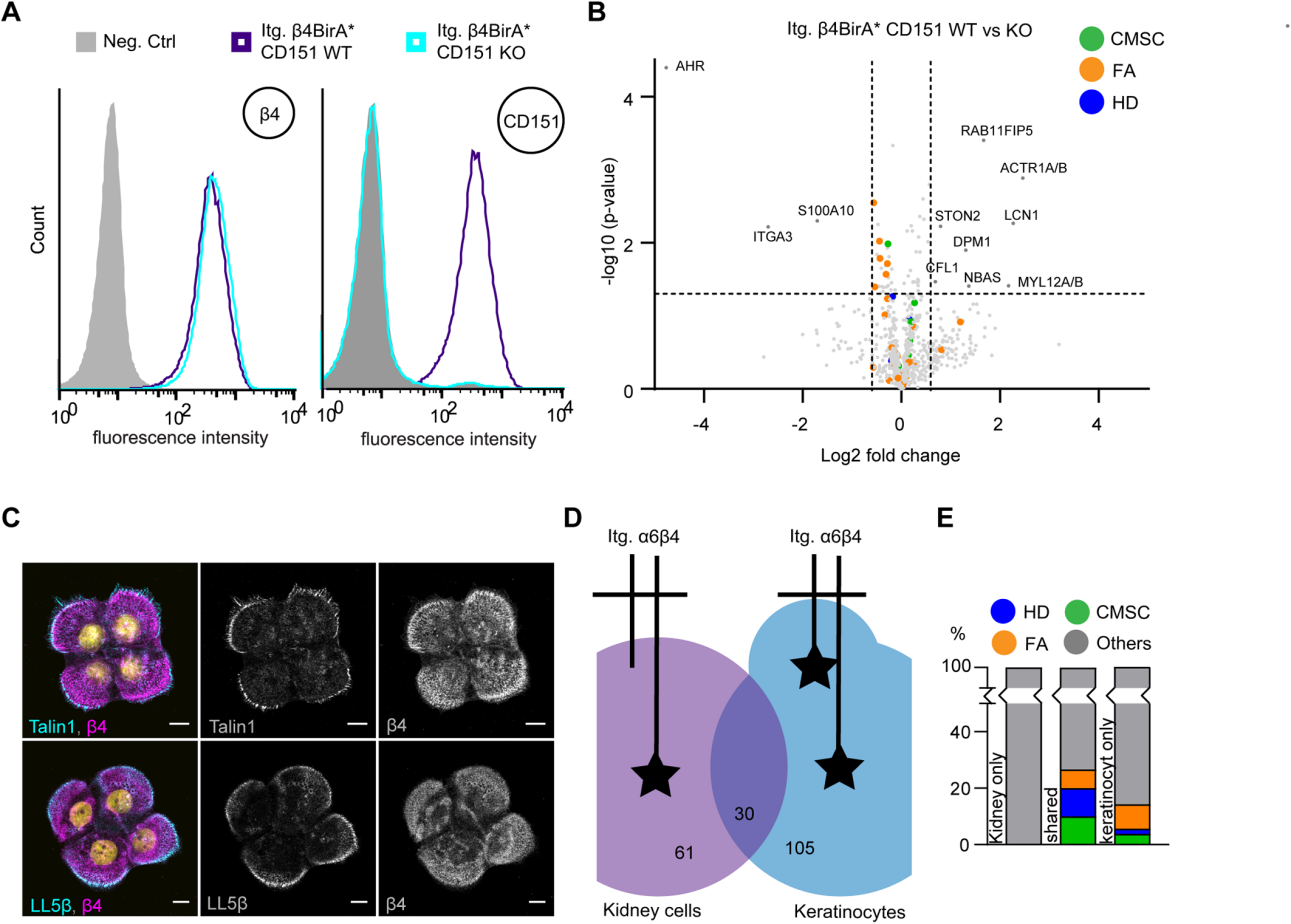
**CMSC and FA proximity interaction with type I** **HDs formed at the cells' periphery.** (A) FACS analysis of β4 and CD151 in PA-JEB/β4-BirA* CD151 wild-type and CD151 KO cells. (B) Volcano plot showing β4 proximity interactors in the presence and absence of CD151 (*n*=3). (C) Immunofluorescence analysis of wild-type HaCaT keratinocytes showing colocalization of β4 with LL5β and talin1. Scale bars: 10 μm. (D) Venn diagram comparing the α6β4 interactome in keratinocytes with that of β4 in MDCK cells (Myllymäki et al., 2019). Numbers of overlapping and specific proteins are presented. (E) Bar graph showing the percentage of HD, CMSC and FA proteins among the unique interactors of α6β4 (105) and the β4 (61) in MDCK and PA-JEB/β4 keratinocytes, as well as their percentage among the interactors that are shared between the two interactomes.

Previously, we have shown that α6β4 resides in both central and peripheral adhesions in keratinocytes (te Molder et al., 2019). While the peripheral adhesions have all the hallmarks of type I HDs, the central adhesions are more similar to type II HDs but are not associated with keratin filaments and, additionally, contain integrin α3β1. Because CD151 forms a complex with both α3β1 and α6β4, and plays an important role in promoting the formation of the central HD-like adhesions, we wondered whether some of the components of the β4 interactome depend on the presence of CD151. To this end, we generated CD151-deficient PA-JEB/β4-BirA* keratinocytes by CRISPR/Cas9-mediated gene disruption and subjected them to BioID. Deletion of CD151 was confirmed by FACS analysis (Fig. 3A). BioID results showed that the β4 interactomes in CD151-proficient and -deficient keratinocytes overlapped each other almost completely; only 11 different β4 interactors were identified (Fig. 3B Table S4). Integrin α3β1, a CD151 binding partner was significantly more associated with β4 in the presence of CD151 than in its absence. Additionally, several proteins associated with RhoA/ROCK-mediated contractility and trafficking were associated with β4 specifically in the presence or absence of CD151. Importantly, none of the identified proteins were components of HDs, CMSCs or FAs (Fig. 3B; Table S4). These results show that despite the fact that CD151 forms a complex with α6β4, this protein does not substantially contribute to the α6β4 interactome. Furthermore, these findings suggest that the CD151-containing adhesions in the central region of the cells hardly contribute to the interaction between HDs and CMSCs or FAs. Indeed, IF analysis in HaCaT keratinocytes revealed that CMSCs and FAs are assembled in close association with the peripheral HDs, but not the central tetraspanin adhesion structures (Fig. 3C).

In simple epithelial tissues and cell lines, such as intestinal and mammary gland cells, integrin α6β4 typically resides in type II HDs (Uematsu et al., 1994; Fontao et al., 1997). The group of Aki Manninen used BioID to identify 91 significant cytoplasmic proximity interactors of α6β4 in Madin Darby Canine kidney epithelial cells (Myllymäki et al., 2019)*.* We compared these interactors with the interactors identified for α6β4 in keratinocytes and found that only 30 proteins were common between the two data sets (Fig. 3D; Table S5). Although more components of cell matrix complexes were found in keratinocytes than in kidney cells, many of the common hits were CMSC, FA and HD members. These results suggest that the interaction between these complexes is not restricted to type I HDs but also occurs at type II HDs (in kidney cells).

### CMSCs are not required for the formation of HDs and vice versa

**Fig. 4.**
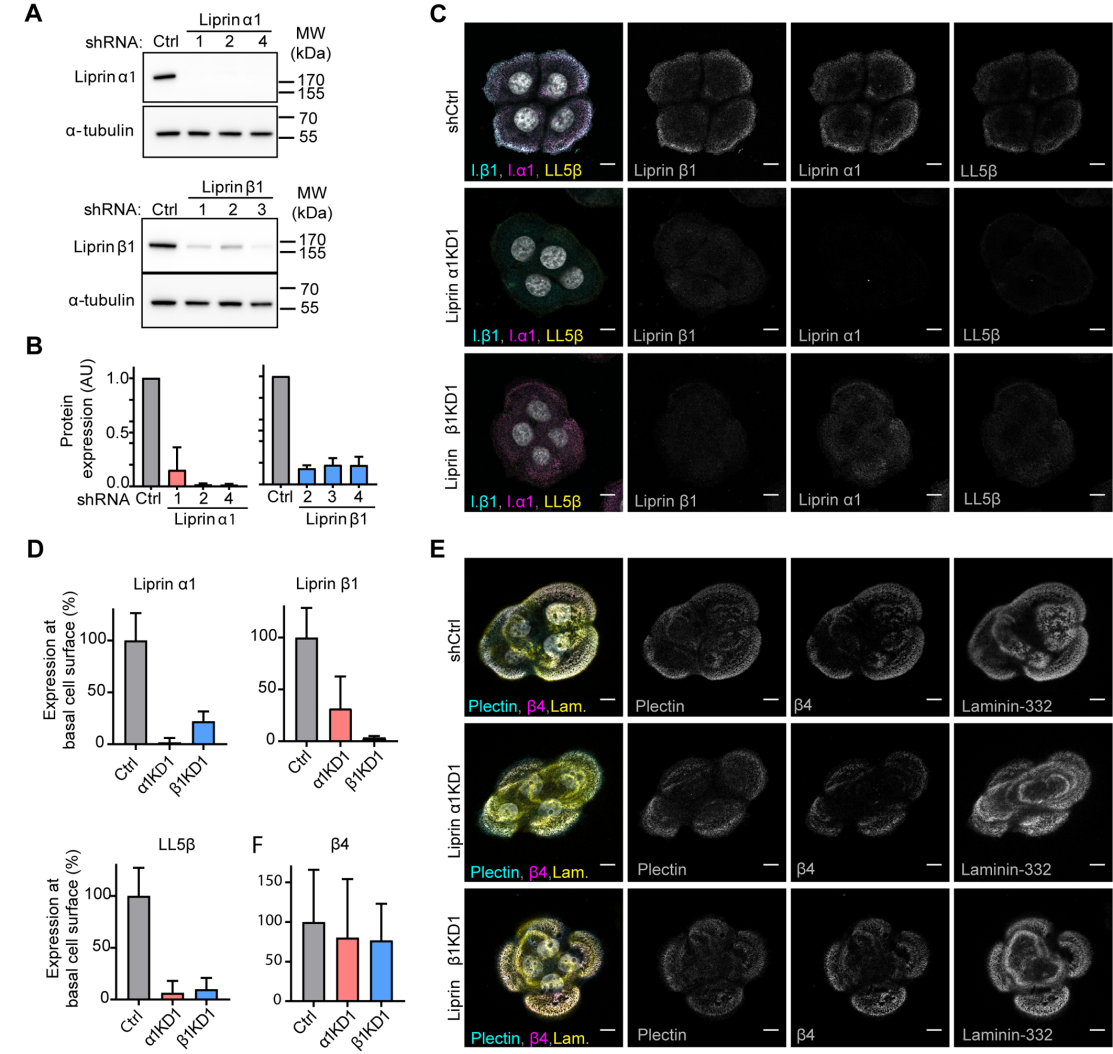
**CMSCs are not required for the formation of HDs in keratinocytes.** (A) Western blot analysis of stable shRNA-expressing PA-JEB/β4 cell lines [control (Ctrl) and three knockdowns (KDs)] probed with antibodies against liprin α1, liprin β1 and α-tubulin. (B**)** Quantification of liprin protein expression normalized to α-tubulin protein expression levels in knock down and control PA-JEB/β4 keratinocytes. Mean+s.d., *n*=2. (C**)** Triple immunofluorescence detection of liprin α1, liprin β1 and LL5β in liprin α1 and β1 knockdown and control keratinocytes. Scale bars: 10 μm. (D) Quantification of immunofluorescence staining of liprin α1, liprin β1 and LL5β in knockdown and control PA-JEB/β4 keratinocytes (*n*=20). (E) Triple immunofluorescence detection of β4, plectin and laminin-332 in liprin α1 and β1 knockdown and control PA-JEB/β4 keratinocytes. Scale bars: 10 μm. (F) Quantification of immunofluorescence staining of β4 shows no significant difference (Mann–Whitney test used) between liprin α1 and β1 knockdown and control PA-JEB/β4 keratinocytes (*n*=22).

Our finding that CMSC proteins are found in close proximity of α6β4 containing HDs raises the question whether this complex plays a role in the formation of HDs by providing a platform at the plasma membrane for the delivery of exocytotic vesicles carrying specific HD components. To investigate the contribution of CMSCs in the formation of HDs, we generated stable liprin α1 and β1 knockdown PA-JEB/β4 keratinocytes by short hairpin RNA (shRNA)-mediated RNA interference. Efficient knockdown of these proteins was confirmed by western blot analysis (Fig. 4A,B). Quantification of the protein levels showed that all shRNAs reduced the expression of their targeted proteins by at least 80% (Fig. 4B). Knockdown of liprin α1 or β1 almost completely prevented the formation of CMSCs as judged by immunofluorescence (Fig. 4C,D). However, HD formation, assessed by β4-plectin colocalization, was unaffected by the loss of the CMSCs (Fig. 4E).

**Fig. 5.**
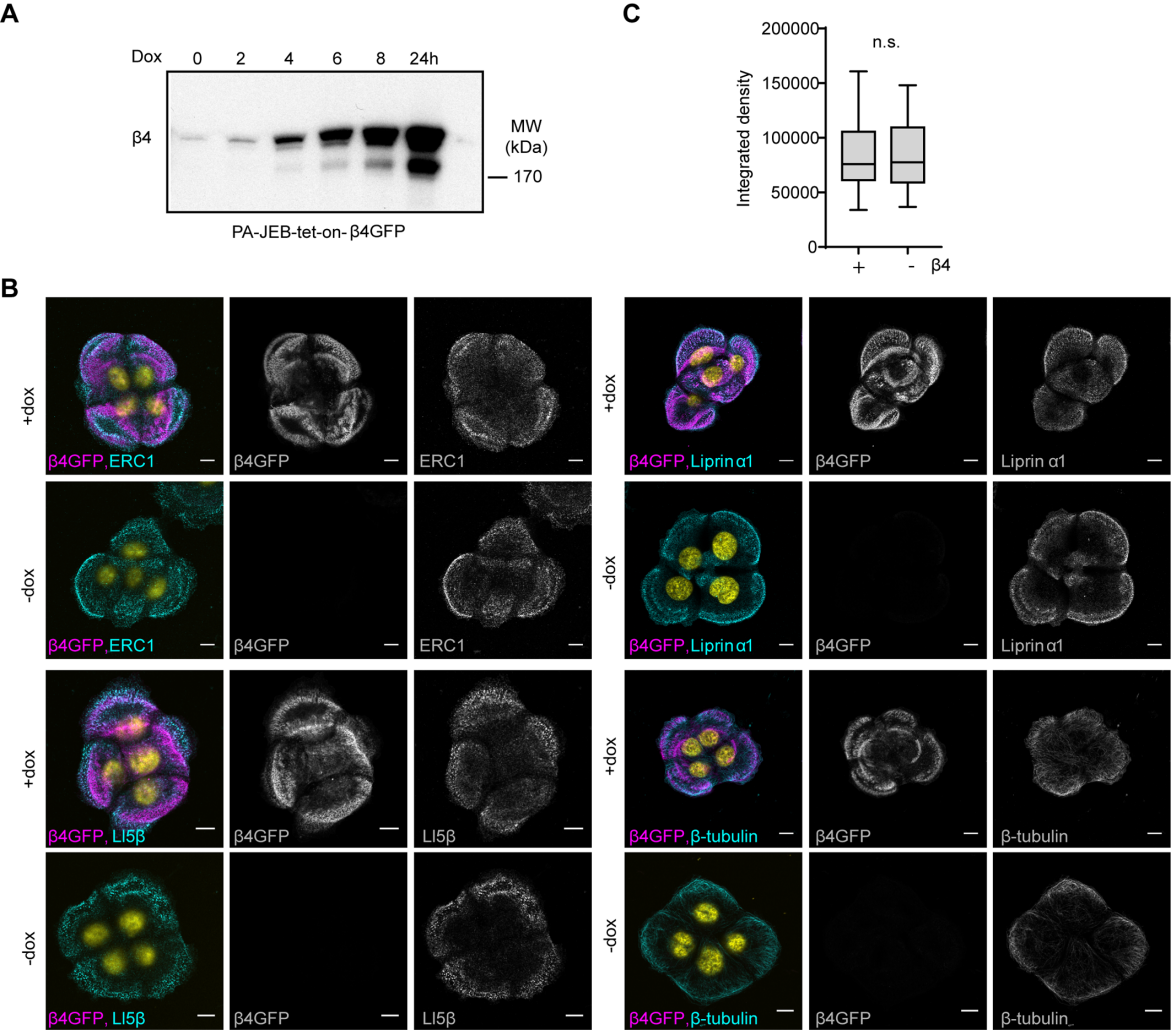
**HDs are not required for the formation of CMSCs.** (A) Western blot analysis of whole cell lysates from PA-JEB-tet-on-β4-GFP cells treated for different time points with doxycycline, and analyzed for β4. (B) Immunofluorescence analysis of CMSC members in PA-JEB keratinocytes treated with (+) or without (−) doxycycline. Cells were stained for ERC1, liprin α1, LL5β or β-tubulin. Scale bars: 10 μm. (C) Quantification of membrane associated liprin α1, integrated density (area*intensity), in PA-JEB-tet-on-β4-GFP treated with or without doxycycline for 24 h (*n*=20). No significant difference; tested with Mann–Whitney test.

To investigate if, conversely, HDs affect the formation of the CMSCs, we compared the presence of CMSCs in the presence or absence of HDs by using PA-JEB keratinocytes that express β4 upon doxycycline induction. The expression of β4 was time-dependent and reached a maximum at 24 h after induction (Fig. 5A). Immunofluorescence analysis of β4-deficient and -proficient cells (assessed after 24 h doxycycline induction) showed no obvious difference in the cellular distribution of CMSC protein localization. Moreover, no difference in the clustering intensities of CMSC proteins were observed (Fig. 5B,C).

In summary, this data show that under steady state conditions the assembly of HDs and CMSCs are not dependent on each other.

## DISCUSSION

In this study, we identified 135 interactors of the integrin α6β4, which include amongst others several FA proteins and all components of the CMSC complex. Recent evidence suggests that CMSCs contribute to FA turnover by facilitating integrin β1 recycling and enabling directional transport of MMPs via microtubules (Asperti et al., 2010; Astro et al., 2014; Mana et al., 2016). In a similar way, CMSCs may regulate the turnover of HDs. Indeed, it has been shown that the recycling of α6β4 is dependent on Arf6, which cooperates with microtubules in adhesion-dependent trafficking (Balasubramanian et al., 2007; Osmani et al., 2018). Furthermore, CMSCs might play a role in the directional transport of HD-associated proteins to sites where new HDs are formed. In agreement with this hypothesis, basal microtubules have been shown to control the integrity of the basement membrane in avian development (Nakaya et al., 2008), and CMSCs are located at substratum adhesion sites composed of deposited laminin-5 and laminin-binding integrins (Hotta et al., 2010). It is perhaps interesting to mention here, that MTs have also been implicated in the assembly of desmosomes, intercellular junctions, which like HDs, are anchored to the intermediate filament system. The adhesion strength of desmosomes is dramatically weakened in the absence of the MT motor proteins kinesin-1 and kinesin-2, despite the fact that the desmosomal plaque proteins are not affected (Nekrasova et al., 2011).

However, despite these findings and the fact that CMSCs are found in close proximity of type I HDs in the periphery of the cells, as determined by immunofluorescence and BioID, no defects in HD assembly were observed in keratinocytes depleted of liprin α1 or liprin β1, which causes the disruption of the CMSC. Likewise, the depletion of LL5 in MCF-10A cells did not affect the basal localization of α6β4 (Hotta et al., 2010). However, because our investigations involve the analysis of HDs under steady-state conditions, subtle differences in the dynamics and/or the rate of HD maturation may have been unnoticed. Furthermore, it is possible that the loss of CMSC function is compensated for by other proteins that can anchor MTs at the plasma membrane or the actin cortex (Kodama et al., 2003; Wen et al., 2004; Akhmanova and Hoogenraad, 2005; Wickström et al., 2010; Sakamoto et al., 2012; Moffat et al., 2017). These proteins include APC, IQGAP and ACF7 (also known as MACF1), but none of them were identified in the interactome of the integrin α6β4.

The localization of CMSCs in the immediate vicinity of type I HDs raises the question of whether these complexes are linked to each other. As far as we are aware, there are no CMSC-associated proteins identified that can link CMSCs directly to HDs. This in contrast to the interaction between CMSCs and FAs, which is mediated by the binding of KANK to talin in FAs (Bouchet et al., 2016). Recently, we showed that in keratinocytes, FAs and HDs are localized in close proximity to each other and that plectin plays a critical role in the mechanical coupling of these two adhesion structures (Wang et al., 2020). Hence, we believe that the localization of CMSC near HDs is due to the interaction of CMSC with the HD-associated FAs.

Comparison of the β4 interactomes in keratinocytes and MDCK cells revealed an overlap of only 30 interactors. CMSC members were present in both interactomes. However, while in the keratinocytes all CMSC members were identified, in the kidney cells only LL5α/β and ERC1 were identified. This relatively small overlap in the two interactomes might be explained by the use of different cell lines and/or the subcellular localization of integrin α6β4 in type I versus type II HDs (Myllymäki et al., 2019). However, it could also have a technical reason, since it has been shown that proteomes identified by different hands and methods can differ considerably (Horton et al., 2016). The approach employed by us to determine the β4 interactome differed from the one used by Myllymäki et al. (2019) in that they used a myristoylated C-terminally BirA*-tagged GFP construct as a negative control, while we used an IL2R-BirA* construct. Furthermore, we expressed the β4-BirA* fusion protein in cells that are deficient in β4, which exclude possible competition between the fusion protein and endogenous β4 for expression at the cell surface because of the pairing of the α6 subunit with β4. Importantly, although the two β4 interactomes differ considerably, CMSC, FA and HD proteins were found as interactors of α6β4 in both datasets, suggesting that the association of CMSCs, FAs and HDs is not unique to keratinocytes. Besides the identification of the CMSC and FA proteins as proximity interactors of integrin α6β4, we observed several proteins that link α6β4 to cell–cell junctions, such as Discs Large Homolog 1 and 5, Afadin and ERBIN. These interactions might occur by a pool of α6β4 molecules that are not present in HD-like adhesions but are located at the lateral aspects of cells. ERBIN, which associates with p0071 at cell–cell junctions, has previously been shown to also bind the integrin β4 subunit (Favre et al., 2001; Izawa et al., 2002).

Intriguingly, we also identified the palmitoyl acyltransferase ZDHHC5 as a proximity interactor of the α6 and β4 subunits. Previous studies have shown that in addition to CD151, both α6 and β4 subunits are also palmitoylated (Yang et al., 2004). The enzyme responsible for the palmitoylation of the α6 and β4 subunits in MDA-MB-231 and PC3 cells has been identified as ZDHHC3 (Sharma et al., 2012). Unfortunately, ZDHHC5 has not been tested for its ability to palmitoylate the α6 and β4 subunits; thus, the possibility exists that this enzyme palmitoylates these subunits in a functional redundant manner. Since palmitoylation stabilizes tetraspanin–tetraspanin interactions as well as their interaction with partner molecules, the palmitoylation of the α6 and β4 subunits may contribute to their stable incorporation into CD151 TEMs that are formed in the central region of the cells.

Although only 11 statistically significant differences were detected between the interactomes of β4 in the presence and absence of CD151, some of them deserve particular attention because they provide further insights into the role of this tetraspanin in the formation and stabilization of the different α6β4-containing adhesions. For example, the identification of integrin α3β1 as a CD151-dependent interactor of integrin α6β4 supports the recently proposed role of CD151 in the clustering of α3β1, together with α6β4, in central HD-like adhesions (te Molder et al., 2019). Furthermore, the identification of two downstream effectors of the RhoA-ROCK signaling pathway, the myosin regulator light chains 12A/B (MYL12A/B) and cofilin (CFL1), as proximity interactors of β4 in the absence of CD151 is in line with the proposed role of CD151 in regulating RhoA activation (Johnson et al., 2009). Indeed, it has been shown that deletion of CD151 results in elevated RhoA activity (Johnson et al., 2009). CD151 may regulate RhoA activity by preventing α3β1 from becoming incorporated into FAs and thus to support high actomyosin contractility of cells cultured on laminin substrates. The finding that the myosin regulator light chains 12A/B and cofilin are CD151-dependent proximity interactors of integrin β4 may seem odd at first sight, but can be explained by the increased FA size and the close proximity of these FAs with HDs in the absence CD151. The ability of CD151 to suppress RhoA activity suggests that CD151 may also contribute to α6β4's ability to counteract cellular tension and traction force in keratinocytes (Wang et al., 2020). Other β4 interactors in the absence of CD151 include several proteins involved in vesicle transport, which suggest that the α6β4 containing adhesions turn over more dynamically in the absence of CD151 than in its presence.

In conclusion, we show that the integrin α6β4 interactome comprises 135 proteins, and includes amongst others FA proteins and components of the CMSC complex, but also many novel proteins that offer opportunities for future research into their role in the assembly of the different α6β4 adhesions.

## MATERIALS AND METHODS

### Reagents

The primary antibodies used in this study are listed in Table 1. Conjugated antibody against biotinylated proteins was streptavidin-HRP (RPN1231 from GE Healthcare). Secondary antibodies for western blot were goat anti-mouse IgG HRP (Bio-Rad; 1:3000) and polyclonal goat anti-rabbit IgG HRP (Dako; 1:5000). Secondary antibody for FACS was donkey anti-mouse IgG PE (Jackson ImmunoResearch; 1:400). Secondary antibodies for IF (1:200) were goat anti-guinea pig IgG Alexa Fluor 488 (A-11073), goat anti-rabbit Alexa Fluor 647 (A-21245), goat anti-rabbit Alexa Fluor 594 (A-21207), goat anti-rat-TxR (T-6392) and goat anti-mouse Alexa Fluor 647 (A-21236) from Invitrogen, goat anti-mouse FITC (Rockland; 610-102-121) and Hamster-Cy5 (Jackson ImmunoResearch; 107-005-142).

**Table 1.**
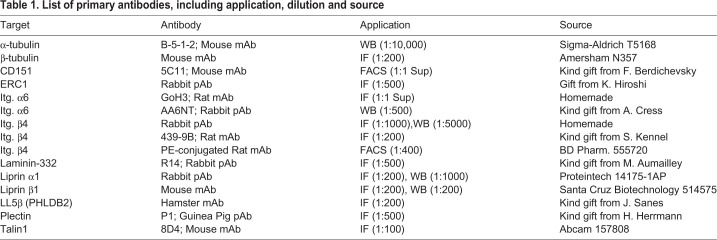
**List of primary antibodies, including application, dilution and source**

### Cell culture

PA-JEB immortalized keratinocytes were isolated from a patient with Pyloric Atresia associated with Junctional Epidermolysis Bullosa. PA-JEB/β4 keratinocytes stably expressing wild-type β4 were generated by retroviral transduction (Sterk et al., 2000) and maintained in serum-free keratinocyte medium (KGM; Invitrogen), supplemented with 50 µg ml^−1^ bovine pituitary gland extract, 5 ng ml^−1^ EGF, and antibiotics (100 units ml^−1^ streptomycin and 100 units ml^−1^ penicillin; Sigma-Aldrich). HaCaT keratinocytes (obtained from the American Type Culture Collection) were cultured in Dulbecco's modified Eagle's medium (DMEM; Gibco) containing 10% heat-inactivated fetal calf serum (FCS; Serana Europe GmbH, Pessin, Germany), and antibiotics. All cells were cultured at 37°C in a humidified, 5% CO_2_ atmosphere.

### Cell culture and generation of stable cell lines

Cell lines stably expressing teton-β4-GFP, β4-BirA*, α6-BirA* or IL2R-BirA* we generated by retrovirus-mediated transduction. Virus was produced by transient transfection of Phoenix-ampho cells with retroviral vector constructs using calcium phosphate precipitation and added to the cells. Cells expressing the protein of interest were enriched by puromycin or zeocin selection and bulk sorted by FACS. The tetON-β4-GFP construct was cloned into the EcoRI site of pRetroX-Tight-Puro vector. The β4-BirA*, α6-BirA* and the IL2R-BirA* constructs used were cloned into the SnaBI site or HindIII/XbaI sites of the LZRS-IRES-Zeo vector.

PA-JEB/β4 α6KO or CD151KO cells were prepared using CRISPR-Cas9 technology. Target sgRNAs against integrin α6 (5′CGGTCGCGAGCTGCCCGCGA3′) or CD151 (5′-CAGGTTCCGACGCTCCTTGA-3′) were cloned in the pX330-U6-Chimeric_BB-CBh-hSpCas9 (Addgene plasmid #42230, deposited by Feng Zhang). The cells were transiently transfected with this plasmid using lipofectamine^®^ 2000 (Invitrogen) in OptiMEM (Gibco), and bulk sorted, for the negative population, using a Moflo Asterios (Beckman Coulter) or FACSAria Fusion (BD Biosciences) cell sorter.

Liprin α1 and liprin β1 were depleted by shRNA-mediated KD in PA-JEB/β4 cells. Recombinant lentiviruses were produced by transient co-transfection of HEK293T cells with plasmids encoding the vector and helper functions using lipofectamine 2000. Lentiviruses were added to the cells and infected cells were enriched by puromycin selection for 3 days. The shRNAs targeted: liprin α1: (1) 5′-GATGACAAGACAACCATAAAG-3′, (2) 5′-TGAGCCTTCCAAGGTACAAAC-3′, (3) 5′-GAGGAGATTGAAAGTCGAGTT-3′; Lirpin β1: (1) 5′-CATTGGCCTCCCTCAATATAA-3′, (2) 5′-CCAGAGTGTTTCCATTCATAT-3′, (3) 5′-GCCAAAGTGAAGCCAAAGAAA-3′ and the empty vector was used as negative control.

### Western blot analysis

Subconfluent cells were washed in cold PBS, lysed in RIPA (1% NP-40, 0.5% sodium deoxycholate, 0.1% SDS, 4 mM EDTA pH 7.5, 100 mM NaCl, 20 mM Tris-HCl pH 7.5) supplemented with 1.5 mM Na_3_VO_4_, 15 mM NaF as phosphatase inhibitors and a protease inhibitor cocktail (1:500; Sigma-Aldrich). Cell lysates were cleared by centrifugation at 12,000 rpm, 1 h at 4°C, supplemented with SDS sample buffer (50 mM Tris-HCl pH 6.8, 2% SDS, 10% glycerol, 12.5 mM EDTA, 0.02% Bromophenol Blue) with β-mercaptoethanol and heated for 5 min at 95°C. Proteins were separated on 4–12% bolt gradient gels (Invitrogen) and transferred to Immobilon-P transfer membranes (Millipore). The membrane was blocked for 3 h in 2% BSA in TBST (10 mM Tris (pH 7.5), 150 mM NaCl, 0.05% Tween 20) before incubation with primary antibody overnight at 4°C and with secondary antibody for 1 h at room temperature or with conjugated Ab for 4 h. After each incubation step, the membranes were washed twice with TBST and twice with TBS (TBST without Tween 20). Antibodies were detected using Clarity Western ECL Substrate (Bio-Rad). Quantification of protein expression was performed on two independent experiments using ImageJ.

### Flow cytometry analysis

Subconfluent cells were trypsinized and collected in PBS supplemented with 2% FCS. Cells were incubated for 50–60 min with primary antibody on ice, washed twice with ice-cold PBS/2% FCS and subsequently incubated with secondary antibody for 50 min on ice. After washing with 2% FCS in PBS, the cells were passed through a nylon mesh filter and 50,000 positive cells were analyzed per sample using a FACSCalibur cell analyzer (BD Biosciences). Unstained cells or cells treated only with secondary antibody were used as negative controls.

### Immunofluorescence

Cells were grown on uncoated coverslips for 20 h before changing the medium to DMEM supplemented with 10% FCS to facilitate HD formation. 20 h after changing the medium the cells were washed with PBS, fixed with 2% PFA for 10 min, permeabilized with 0.2% triton for 5 min and blocked with 2% BSA (Serva) in PBS for 1 h. Cells were incubated with primary and secondary antibodies for 50–60 min each with PBS washing steps in between. Nuclei were stained with DAPI for 20 s and coverslips were mounted on slides using MOWIOL. After drying the slides overnight, cell islands of four cells were imaged using a Leica TCS SP5 confocal microscope with a 63× objective. Quantification of the intensity, integrated density/expression at the basal cell surface in 20–22 islands was performed using ImageJ.

### BioID sample preparation

Cells were seeded for 24 h in biotin-depleted keratinocyte medium before being treated with 50 μM biotin (Sigma-Aldrich #B4501) for 20 h in DMEM/10% FCS. After washing with cold PBS, cells were lysed at 4°C in NP40 lysis buffer (1% Nonidet P-40, 20 mM Tris-HCl, pH 7.5, 100 mM NaCl, and 4 mM EDTA) supplemented with Na_3_VO_4_ (1.5 mM), NaF (15 mM) and protease inhibitor cocktail (1:1000; Sigma-Aldrich). Collected whole cell lysates were cleared by centrifugation at 12,000 rpm for 60 min at 4°C and cleared cell lysates were incubated with Streptavidin Sepharose High Performance beads (GE Healthcare) overnight at 4°C. After incubation beads were washed with NP40 lysis buffer, cold PBS and beads were stored at −80°C.

### Mass spectrometry analysis

Peptide mixtures were prepared from three replicate experiments, measured and analyzed as previously described (Wang et al., 2020), with the following exceptions. The MS/MS data were searched against the human SwissProt database (20,367 entries, release 2020_01) complemented with a list of common contaminants and concatenated with the reversed version of all sequences. Differentially expressed proteins were determined using a Student's *t*-test (minimum threshold *P*≤0.05 and [x/y]≥0.6|[x/y]≤−0.6).

## Supplementary Material

Supplementary information
