# Supplementary material for: Comparative interactomics analysis reveals potential regulators of α6β4 distribution in keratinocytes
Source: Biol Open. 2020 Aug 13;9(8):bio054155. doi: 10.1242/bio.054155 (PMC7438003; doi:10.1242/bio.054155)
Supplement: Supplementary information [file biolopen-9-054155-s1.pdf]

Table S1 β4-BirA* minus versus plus biotin (n=3)

Hit nr.	gene_name	-Log Student's T-test p-value	Student's T-test Difference	Significant or Not Significant
1	**C6orf132**	4,983474	5,829441	Significant
2	**STRAP**	5,358323	5,439143	Significant
3	**DVL3**	4,667804	4,740303	Significant
4	**PHLDB2**	2,767684	6,536439	Significant
5	**KANK2**	2,87541	5,658972	Significant
6	**TJP1**	2,212647	6,15713	Significant
7	**PRAG1**	2,468922	5,618502	Significant
8	**ERBIN**	2,658173	5,390096	Significant
9	**PHLDB1**	2,960457	4,994458	Significant
10	**DLG5**	2,553412	4,98373	Significant
11	**KANK1**	2,843031	4,506907	Significant
12	**TMEM209**	3,98492	3,294238	Significant
13	**SARG**	2,307854	4,831307	Significant
14	**RASAL2**	3,131187	3,974478	Significant
15	**TANC1**	1,745995	5,081182	Significant
16	**NUMB**	1,739105	4,998629	Significant
17	**ARHGAP32**	1,588253	5,083693	Significant
18	**CASKIN2**	2,656641	3,954532	Significant
19	**AFDN**	2,627567	3,92501	Significant
20	**PPFIBP1**	2,574133	3,954645	Significant
21	**HCFC1**	3,443631	3,035116	Significant
22	**ZDHHC5**	2,078496	4,391307	Significant
23	**ITGB4**	2,750032	3,707239	Significant
24	**CKAP5**	2,024366	4,387561	Significant
25	**KIAA1217**	2,076192	4,219676	Significant
26	**SEPTIN10**	3,578716	2,683586	Significant
27	**DLG1**	2,375055	3,864595	Significant
28	**TNS3**	2,848121	3,311089	Significant
29	**PHACTR4**	2,683133	3,387091	Significant
30	**XRN1**	1,963038	4,076722	Significant
31	**DST**	3,602855	2,43597	Significant
32	**PXN**	2,098293	3,880695	Significant
33	**ALMS1**	3,078797	2,887848	Significant
34	**EGFR**	2,443831	3,518398	Significant
35	**TJP2**	2,417764	3,532287	Significant
36	**EPHA2**	2,516803	3,399036	Significant
37	**CDKAL1**	2,254826	3,602667	Significant
38	**USP15**	2,182815	3,664659	Significant
39	**ITGA6**	2,432058	3,38373	Significant
40	**EPB41**	1,6449	4,044989	Significant
41	**BCAR3**	1,683942	3,984955	Significant
42	**COL17A1**	2,650895	3,010379	Significant
43	**SYNJ1**	2,650471	2,985749	Significant
44	**KEAP1**	2,736491	2,898935	Significant

Biology Open • Supplementary information

45	**DBNL**	3,173068	2,459415	Significant
46	**MRE11**	2,61295	2,893466	Significant
47	**CAMSAP1**	2,545257	2,866247	Significant
48	**ZYX**	1,62286	3,758633	Significant
49	**STAT3**	2,489712	2,887028	Significant
50	**OSBPL10**	3,70151	1,671032	Significant
51	**ANKRD50**	2,270448	3,08513	Significant
52	**PDLIM5**	1,46882	3,875849	Significant
53	**OSBPL3**	1,946815	3,373469	Significant
54	**CTNNB1**	2,339995	2,9701	Significant
55	**DSG2**	2,689629	2,615779	Significant
56	**AFAP1L2**	2,344092	2,934981	Significant
57	**TOR1AIP1**	2,194225	3,076172	Significant
58	**P4HB**	2,929372	2,314976	Significant
59	**KNL1**	2,270656	2,957877	Significant
60	**MARK2**	1,536176	3,664413	Significant
61	**PEAK1**	1,933287	3,252141	Significant
62	**PAK6**	2,944358	2,226367	Significant
63	**PLEKHA5**	2,214435	2,943157	Significant
64	**STX5**	2,172632	2,959009	Significant
65	**FAM83B**	1,816628	3,305831	Significant
66	**EIF4G1**	1,485525	3,621386	Significant
67	**FAM135A**	1,375172	3,718792	Significant
68	**CTNNA1**	1,580063	3,480512	Significant
69	**SEPTIN7**	2,806311	2,132664	Significant
70	**MRTFB**	2,327732	2,584309	Significant
71	**GOLGA5**	1,738443	3,137636	Significant
72	**CEP170**	2,407126	2,445349	Significant
73	**SEC23B**	2,780671	2,039096	Significant
74	**CTNND1**	1,573463	3,240248	Significant
75	**TLN1**	2,453104	2,347322	Significant
76	**LIMD1**	2,297273	2,497599	Significant
77	**TACC1**	1,881098	2,883171	Significant
78	**EPS15**	1,453904	3,303209	Significant
79	**TNKS1BP1**	2,397446	2,351044	Significant
80	**TRIP6**	2,11035	2,607492	Significant
81	**KIAA1522**	1,503754	3,17632	Significant
82	**KIDINS220**	1,578511	3,100849	Significant
83	**BAIAP2**	1,844836	2,79409	Significant
84	**NUMBL**	1,468244	3,166171	Significant
85	**SCRIB**	1,304554	3,27726	Significant
86	**SEPTIN2**	2,879636	1,640544	Significant
87	**PTPN13**	1,36217	3,092864	Significant
88	**ZC3HAV1**	2,580625	1,846071	Significant
89	**SNTB2**	1,740071	2,672489	Significant
90	**DPYSL2**	1,506151	2,894411	Significant
91	**CRYBG1**	2,342753	2,056397	Significant
92	**EPS15L1**	1,8667	2,465309	Significant

Biology Open • Supplementary information

93	**CNOT1**	1,326709	2,93485	Significant
94	**UTRN**	1,366115	2,877391	Significant
95	**STON2**	1,49409	2,704155	Significant
96	**MYOF**	1,481122	2,710726	Significant
97	**PLEKHA1**	1,576795	2,594016	Significant
98	**RPS5**	1,786686	2,372269	Significant
99	**PPP2R3A**	1,35673	2,784499	Significant
100	**CLMN**	1,913424	2,200071	Significant
101	**CPSF3**	1,450164	2,658859	Significant
102	**PAK2; PAK3**	1,332827	2,773766	Significant
103	**SLK**	2,140135	1,964647	Significant
104	**CTTN**	1,596139	2,499079	Significant
105	**TBC1D2**	1,430951	2,650555	Significant
106	**DVL1; DVL1P1**	1,45026	2,621412	Significant
107	**VANGL1**	1,911336	2,158401	Significant
108	**HAUS6**	1,482955	2,578769	Significant
109	**TNS4**	1,969631	2,081828	Significant
110	**PARVA**	1,589847	2,45697	Significant
111	**FASN**	2,194431	1,834911	Significant
112	**SEPTIN9**	1,65478	2,33521	Significant
113	**ERC1**	1,48826	2,450827	Significant
114	**TRIM29**	1,948189	1,97771	Significant
115	**HLCS**	1,831847	2,077721	Significant
116	**WDR44**	2,033889	1,81997	Significant
117	**ARHGAP1**	2,054083	1,798428	Significant
118	**HSPA5**	1,941704	1,891386	Significant
119	**GSDME**	1,477945	2,314721	Significant
120	**PPP1R13L**	1,850449	1,931557	Significant
121	**NDC1**	1,308662	2,438623	Significant
122	**EIF5**	1,369071	2,313273	Significant
123	**SRP68**	1,471446	2,094981	Significant
124	**PREB**	1,521998	1,975349	Significant
125	**GIGYF2**	1,323151	2,128295	Significant
126	**NCKAP1**	1,880176	1,507039	Significant
127	**TOR1AIP2**	1,441381	1,905609	Significant
128	**MIA3**	1,396912	1,868633	Significant
129	**CORO1B**	1,691772	1,521893	Significant
130	**MYO1B**	1,421529	1,647255	Significant

Biology Open • Supplementary information

Table S2 β4-BirA* plus biotin versus IL2R-BirA* plus biotin (n=3)

Hit nr.	gene_name	-Log Student's T-test p-value	Student's T-test Difference	Significant or Not Significant
1	**ERC1**	3,481444857	8,505638123	Significant
2	**TLN1**	3,581817628	7,120169322	Significant
3	**ITGB4**	4,544548974	6,099866867	Significant
4	**PHLDB2**	2,983890001	6,950602849	Significant
5	**DLG5**	3,342639935	6,558958054	Significant
6	**KANK2**	3,480366787	6,220681508	Significant
7	**UTRN**	2,847510042	6,780156453	Significant
8	**ERBIN**	4,176936312	5,378283183	Significant
9	**TNS3**	2,98877645	6,512499491	Significant
10	**FLNA**	3,692615081	5,793609619	Significant
11	**C6orf132**	3,636677727	5,681793213	Significant
12	**MRTFB**	3,459058067	5,470768611	Significant
13	**PPFIBP1**	3,049205187	5,679796219	Significant
14	**PHLDB1**	3,317555626	5,358519872	Significant
15	**PXN**	2,984780434	5,661593755	Significant
16	**AFDN**	3,202498378	5,354756037	Significant
17	**CASKIN2**	2,579636516	5,91941007	Significant
18	**ITGA6**	3,69593834	4,710760752	Significant
19	**FAM135A**	3,076519622	5,296726227	Significant
20	**SNTB2**	3,875892229	4,392897288	Significant
21	**HCFC1**	3,665954974	4,557535807	Significant
22	**RANBP2**	3,45937565	4,640565236	Significant
23	**EPS15**	3,506200691	4,381397247	Significant
24	**AHNAK2**	3,888924626	3,950870514	Significant
25	**RASAL2**	3,289661917	4,471676509	Significant
26	**CTTN**	3,303366787	4,451934179	Significant
27	**TJP1**	1,966626185	5,741708755	Significant
28	**EPS15L1**	2,445331222	5,140254339	Significant
29	**LIMD1**	2,777955382	4,573886871	Significant
30	**SARG**	2,696310705	4,625443776	Significant
31	**FASN**	3,384033954	3,843261083	Significant
32	**PRAG1**	2,241282967	4,90564092	Significant
33	**COPG2**	2,378271653	4,754276276	Significant
34	**KEAP1**	3,415543592	3,704780579	Significant
35	**KNL1**	2,834111549	4,157740911	Significant
36	**PAICS**	2,238095701	4,705089569	Significant
37	**DST**	3,219950901	3,594175339	Significant
38	**HAUS6**	2,631781868	4,105353038	Significant
39	**KIAA1217**	2,182224653	4,530141195	Significant
40	**TANC1**	1,736521966	4,944445928	Significant
41	**CORO1B**	2,068802921	4,605710347	Significant
42	**VCL**	2,352787637	4,258904775	Significant
43	**TJP2**	2,069333299	4,457257589	Significant
44	**COL17A1**	3,675474167	2,851025263	Significant

Biology Open • Supplementary information

45	**ZYX**	3,356762107	3,152051926	Significant
46	**KANK1**	2,690169364	3,815174103	Significant
47	**DLG1**	3,046704133	3,391640981	Significant
48	**DVL3**	2,648674334	3,73213768	Significant
49	**MRE11**	3,082871523	3,254869461	Significant
50	**PDLIM5**	2,817555645	3,458613078	Significant
51	**CCT8**	3,703036455	2,57158788	Significant
52	**ZDHHC5**	2,727467918	3,505296707	Significant
53	**SYNJ1**	2,835113525	3,36527888	Significant
54	**FLNB**	2,547081332	3,589440028	Significant
55	**GIGYF2**	1,935724205	4,193858465	Significant
56	**NUMB**	2,075414634	4,018562317	Significant
57	**CD2AP**	1,98686568	4,100517273	Significant
58	**TNKS1BP1**	3,694091508	2,382957458	Significant
59	**CAMSAP1**	3,217282284	2,838649114	Significant
60	**RAI14**	3,354103418	2,691554387	Significant
61	**XRN1**	2,190858906	3,76820755	Significant
62	**EPB41**	1,803677296	4,113066991	Significant
63	**PPP1R13L**	3,063666472	2,851318359	Significant
64	**BCAR3**	2,380913043	3,505453746	Significant
65	**CRK**	1,882988449	3,882542292	Significant
66	**PPFIA1**	1,996505563	3,761895498	Significant
67	**STON2**	2,173356114	3,577549616	Significant
68	**GEMIN5**	2,404223592	3,230573654	Significant
69	**SUMO1; SUMO1P1**	2,386578667	3,24376742	Significant
70	**PLEKHA5**	2,446916112	3,096908569	Significant
71	**ARHGAP32**	1,561314621	3,890457153	Significant
72	**NIBAN2**	2,13109677	3,285207113	Significant
73	**NUP155**	1,73024664	3,636830012	Significant
74	**NUMBL**	2,353817544	2,903125763	Significant
75	**FLNC**	2,44701986	2,788606008	Significant
76	**PGAM5**	1,592931241	3,64104716	Significant
77	**PEAK1**	2,043743571	3,09951973	Significant
78	**GSDME**	2,469893718	2,571150462	Significant
79	**EZR**	2,494924515	2,540669759	Significant
80	**PLEC**	2,332927231	2,695134481	Significant
81	**SEPTIN11**	1,434980548	3,572987874	Significant
82	**EIF4G1**	1,704669512	3,302465439	Significant
83	**TNS4**	2,125851587	2,849858602	Significant
84	**DBNL**	2,517551864	2,45506223	Significant
85	**SEC23B**	2,674478003	2,275936127	Significant
86	**ANKRD50**	1,986281071	2,940560659	Significant
87	**NUP133**	1,86793902	3,040430069	Significant
88	**USP15**	2,159837345	2,73382314	Significant
89	**DPYSL2**	1,788395045	3,086363475	Significant
90	**DVL1; DVL1P1**	2,594036395	2,095871607	Significant
91	**RANGAP1**	2,486894601	2,142262141	Significant

Biology Open • Supplementary information

92	**SCRIB**	1,344199081	3,262561162	Significant
93	**MYOF**	2,003924906	2,574784597	Significant
94	**AFAP1L2**	2,008192016	2,545626958	Significant
95	**PHACTR4**	2,077948368	2,438491821	Significant
96	**CLMN**	1,786895948	2,628778458	Significant
97	**MYO1B**	2,009816238	2,405704498	Significant
98	**PTPN12**	1,770341919	2,527658463	Significant
99	**KIAA1522**	1,434910617	2,782882055	Significant
100	**TBC1D2**	1,61659234	2,586899439	Significant
101	**KIDINS220**	1,593514253	2,492033005	Significant
102	**TAB1**	1,759756465	2,261226018	Significant
103	**LARP1**	1,735629349	2,248367945	Significant
104	**CNOT1**	1,804821663	2,127309163	Significant
105	**ALMS1**	1,89143119	2,016126633	Significant
106	**EFHD2**	1,530886966	2,349688212	Significant
107	**LAMC2**	1,77275406	2,102012634	Significant
108	**TRIP6**	1,752601881	2,068318685	Significant
109	**AHNAK**	1,796851728	2,004161835	Significant
110	**CKAP5**	1,695504425	2,085919062	Significant
111	**EIF4E2**	1,410038458	2,31280454	Significant
112	**LUZP1**	1,653578751	2,033416748	Significant
113	**STRAP**	1,351146383	2,279120763	Significant
114	**GAPVD1**	1,364759025	2,098588943	Significant
115	**SEC24A**	1,342436151	2,096307755	Significant
N.A.	**IL2RA**	3,032817452	-7,126452764	Significant

Biology Open • Supplementary information

Table S3 α6-BirA* plus biotin versus IL2R-BirA* plus biotin (n=3)

Hit nr.	gene_name	-Log Student's T-test p-value	Student's T-test Difference	Significant or Not Significant
1	**PPFIBP1**	4,669456	6,347483	Significant
2	**KRT6B**	3,111004	7,094639	Significant
3	**ITGA6**	4,288527	5,560982	Significant
4	**ITGB4**	4,775116	4,975218	Significant
5	**CCT8**	3,950828	4,540769	Significant
6	**CASKIN2**	2,869794	5,001071	Significant
7	**PPFIA1**	3,255039	4,556779	Significant
8	**GIGYF2**	2,989378	4,798739	Significant
9	**CPSF1**	4,077145	3,630538	Significant
10	**COL17A1**	3,806056	3,784993	Significant
11	**AFDN**	3,039809	4,351351	Significant
12	**TLN1**	2,91994	4,389297	Significant
13	**FAM135A**	3,383138	3,919304	Significant
14	**RANBP2**	3,08683	3,62579	Significant
15	**FERMT2**	2,455125	4,022923	Significant
16	**ATXN2L**	2,702989	3,616952	Significant
17	**UBAP2L**	2,496555	3,812288	Significant
18	**PXN**	2,954383	3,328231	Significant
19	**TNS3**	1,745012	4,476346	Significant
20	**DLG1**	2,609315	3,538345	Significant
21	**RASAL2**	3,083443	2,994352	Significant
22	**HNRNPK**	3,915844	2,144055	Significant
23	**SNTB2**	2,56236	3,424726	Significant
24	**MATR3**	3,634498	2,304412	Significant
25	**KANK2**	2,45178	3,45656	Significant
26	**UBAP2**	3,573092	2,310243	Significant
27	**FLNA**	3,237366	2,54853	Significant
28	**PHLDB2**	2,359253	3,390733	Significant
29	**PEAK1**	2,468034	3,219858	Significant
30	**FUS**	1,374602	4,165538	Significant
31	**S100A9**	2,112974	3,355374	Significant
32	**PHLDB1**	2,493957	2,838808	Significant
33	**CPSF7**	1,840395	3,39662	Significant
34	**BCL9L**	2,346201	2,765827	Significant
35	**RUVBL1**	2,680651	2,320124	Significant
36	**PLEC**	2,225699	2,706984	Significant
37	**KEAP1**	2,538155	2,375415	Significant
38	**CRK**	1,56615	3,283579	Significant
39	**IGF2BP2**	1,631004	3,077057	Significant
40	**NUMB**	2,042991	2,58675	Significant
41	**SERPINB2**	2,569307	2,052409	Significant
42	**EPS15L1**	1,835766	2,744539	Significant
43	**LAMC2**	1,748527	2,822986	Significant
44	**RUVBL2**	2,245734	2,261399	Significant

Biology Open • Supplementary information

45	**PPP1R13L**	2,186067	2,316207	Significant
46	**ZDHHC5**	2,065901	2,403255	Significant
47	**ERBIN**	2,006092	2,428099	Significant
48	**XRN1**	1,596127	2,833532	Significant
49	**MRTFB**	1,438106	2,841951	Significant
50	**RBM27**	1,802634	2,413303	Significant
51	**DVL1; DVL1P1**	1,981644	2,080001	Significant
52	**FAM83H**	1,476162	2,473873	Significant
53	**NIBAN2**	1,802916	2,117793	Significant
54	**DLG5**	1,642364	2,168767	Significant
55	**FUBP3**	1,592617	2,172834	Significant
56	**TJP2**	1,45394	2,308683	Significant
57	**SNX1**	1,402968	2,278321	Significant
N.A.	**IL2RA**	2,721453	-6,57328	Significant
N.A.	**ITGB1**	2,062781	-2,60988	Significant

Table S4 β4-BirA* plus biotin CD151 proficient vs deficient (n=3)

Hit nr.	gene_name	-Log Student's T-test p-value	Student's T-test Difference	Significant or Not Significant
1	**ACTR1A; ACTR1B**	2,883732626	2,465718587	Significant
2	**RAB11FIP5**	3,402613417	1,666626612	Significant
3	**LCN1**	2,268364451	2,268799464	Significant
4	**MYL12A; MYL12B**	1,40990162	2,174990336	Significant
5	**DPM1**	1,89853465	1,309476217	Significant
6	**STON2**	2,226312941	0,796698252	Significant
7	**NBAS**	1,408316234	1,369881312	Significant
8	**CFL1**	1,467073348	0,689799627	Significant
9	**S100A10**	2,301280868	-1,701531092	Significant
10	**AHR**	4,39578979	-4,764198303	Significant
11	**ITGA3**	2,218476443	-2,697850545	Significant

Biology Open • Supplementary information

Table S5 Identified α6β4 interactome in keratinocytes versus hβ4-BirA interactors in MDCK cells*

α6β4 interactors in keratinocytes	Common	β4 interactors in MDCK cells
AFAP1L2	AHNAK	37500
AFDN	AHNAK2	ACSL3
ALMS1	CCT8	ALDH3A2
ANKRD50	CD2AP	ANKLE2
ARHGAP32	COL17A1	ARHGEF12
ATXN2L	COPG2	C2CD2
BCAR3	CORO1B	CAPZB
BCL9L	CRK	CCT2
C6orf132	CTTN	CCT3
CAMSAP1	EIF4G1	CCT4
CASKIN2	ERC1	CCT6A
CKAP5	HCFC1	CCT7
CLMN	ITGA6	CKAP4
CNOT1	ITGB4	CLCC1
CPSF1	KIAA1217	COPG1
CPSF7	NUP133	CSDE1
DBNL	PDLIM5	DSG2
DLG1	PHLDB1	EIF2AK3
DLG5	PHLDB2	EIF5
DPYSL2	RANBP2	EMC1
DST	RANGAP1	EMD
DVL1; DVL1P1	RUVBL1	EML4
DVL3	RUVBL2	ESYT1
EFHD2	SEC23B	EVPL
EIF4E2	STRAP	FAM91A1
EPB41	TJP1	GRAMD3
EPS15	TJP2	INF2
EPS15L1	TNKS1BP1	JUP
ERBIN	TNS3	KPNB1
EZR	UTRN	KTN1
FAM135A		LBR
FAM83H		LRBA
FASN		MIA3
FERMT2		MOSPD2
FLNA		NAP1L4
FLNB		NUP214
FLNC		NUP62
FUBP3		OSBPL11
FUS		PDAP1
GAPVD1		PDZD8

Biology Open • Supplementary information

GEMIN5
GIGYF2
GSDME
HAUS6
HNRNPK
IGF2BP2
KANK1
KANK2
KEAP1
KIAA1522
KIDINS220
KNL1
KRT6B
LAMC2
LARP1
LIMD1
LUZP1
MATR3
MRE11
MRTFB
MYO1B
MYOF
NIBAN2
NUMB
NUMBL
NUP155
PAICS
PEAK1
PGAM5
PHACTR4
PLEC
PLEKHA5
PPFIA1
PPFIBP1
PPP1R13L
PRAG1
PTPN12
PXN
RAI14
RASAL2
RBM27
S100A9
SARG
SCRIB
SEC24A
SEPTIN11
PPL
RANBP3
RDX
RRBP1
SEC13
SEC16A
SEC24B
SgK223
SHROOM2
SRPR
SSFA2
STIM1
TACC1
TCP1
TMPO
TOR1AIP1
UBXN4
VAPA
VAPB
VCPIP1
WDR11

Biology Open • Supplementary information

SERPINB2
SNTB2
SNX1
STON2
SUMO1; SUMO1P1
SYNJ1
TAB1
TANC1
TBC1D2
TLN1
TNS4
TRIP6
UBAP2
UBAP2L
USP15
VCL
XRN1
ZDHHC5
ZYX

***Myllymäki et al. MCP 2019; Table S1; Proteins that were more that 3-fold enriched in hβ4-BirA samples relative to the BirA-negative control**

Biology Open • Supplementary information
